# New Appliances and Things Medical

**Published:** 1897-03-27

**Authors:** 


### NEW APPLIANCES AND THINGS MEDICAL.

**[We shall be glad to receive, at our Office, 28 & 29, Southampton Street, Strand, London, W.C., from the manufacturers, specimens of all new preparations and appliances which may be brought out from time to time.]**

### THE "ECLIPSE" THERMOMETER.

(S. Maw, Son, and Thompson, 7-12, Aldersgate Street, E.C.)

A new thermometer, which is now being sold by the above firm, is called the "Eclipse." It has several novel features that are likely to be highly appreciated by doctors, nurses, and patients. The scale is cut into the enamel at the back of the tube, and is therefore practically indelible, and the markings are further rendered evident by blacking. The anterior surface of the tube is more or less angular, and acts as a magnifying glass for the column of mercury and the scale. The posterior surface is flattened, and, consequently, the instrument cannot roll off the table and become broken. We have made practical trial of this new thermometer, and find it accurate, and very handy for clinical use, and hence can confidently recommend it to the notice of our readers.

### POND'S ARCH SUPPORTS.

(J. Pond, Castle Meadow, Norwich.)

We have on a former occasion made mention of these excellent arch supports which are intended to replace the usual valgus pads, and alleviate the discomforts of flat foot. They are made in the form of an artificial sole to wear inside the boot, with a well-shaped elevation on the inner side to support the arch and correct any tendency to flattening. They have been well received, especially by members of the nursing profession, and by others who from constitutional weakness or long hours of standing find that the ligaments of the natural arch give way under the strain. To meet an increasing demand among more humble members of society who cannot afford the highly finished article, the above firm are reproducing the arch support in a cheaper form, and it is to the cheaper variety that we wish to call the attention of our readers.

